# Topoisomerase II alpha expression and the benefit of adjuvant chemotherapy for postoperative patients with non-small cell lung cancer

**DOI:** 10.1186/1471-2407-10-621

**Published:** 2010-11-10

**Authors:** Shi Yan, Jiao Shun-Chang, Chen Li, Li Jie, Lv Ya-Li, Wang Ling-Xiong

**Affiliations:** 1 Department of Medical Oncology, General Hospital of CPLA, No.28 FuXing Road, Beijing, China; 2 Department of Pathology, General Hospital of CPLA, No.28 FuXing Road, Beijing, China; 3 Cancer Center Laboratory, General Hospital of CPLA, No.28 FuXing Road, Beijing, China

## Abstract

**Background:**

Adjuvant chemotherapy has been shown to improve survival rates of postoperative patients with non-small cell lung cancer (NSCLC). Biomarkers could help select an appropriate chemotherapy for NSCLC patients or predict the efficacy of chemotherapy. The objective of this study was to explore the possible prognostic and predictive role of topoisomerase II alpha (TopIIα) expression level in postoperative NSCLC patients who received adjuvant chemotherapy.

**Methods:**

Patients with stage I-III NSCLC, who underwent surgery in our hospital from January 2004 to December 2007 and who also received adjuvant chemotherapy after surgery, were analyzed in this study. Expression of TopIIα and Ki67 in paraffin-embedded tissues was detected by immunohistochemistry (IHC). The relationships between clinicopathological characteristics, chemotherapy regimens, the expression of biomarkers and disease free survival (DFS) were analyzed.

**Results:**

TopIIα and Ki67 were highly expressed in 22.5% and 36.4% of the 151 patients, respectively. Univariate survival analysis showed that male sex (P = 0.036), non-adenocarcinoma (P = 0.004), earlier pathological TNM stage (P = 0.001) or pathological N stage (P < 0.001), and high expression of TopIIα (P = 0.012) were correlated with better DFS, whereas age, smoking history, different chemotherapy regimens, T stage and expression level of Ki67 were of no prognostic significance. Further stratified analysis showed that vinorelbine (NVB)-containing adjuvant regimens were generally associated with better DFS than regimens without NVB in patients with low TopIIα expression, though the difference was not statistically significant (P = 0.065). Pairwise comparisons for patients with low TopIIα expression indicated that the NVB-containing regimen was associated with better DFS than the docetaxel (TXT)-containing regimen (P = 0.047). COX multivariate analysis showed that pathological TNM stage, histological subtype and expression level of TopIIα to be independent of risk factors affecting DFS in postoperative NSCLC patients who received chemotherapy.

**Conclusions:**

High TopIIα expression was discovered to be correlated with better DFS for postoperative NSCLC patients who received adjuvant chemotherapy. The NVB-containing chemotherapy regimen was more effective than the TXT-containing regimen in improving DFS in patients with low TopIIα expression. TopIIα could be considered to be an independent prognostic biomarker of DFS in postoperative NSCLC patients who received adjuvant chemotherapy.

## Background

Non-small cell lung cancer (NSCLC) remains one of the most common cancers in China and in the world [1]. Although surgery is the main curative treatment, postoperative recurrence ranges from 30% to 50% [2]. Positive results of recent clinical trials, including the International Adjuvant Lung Cancer Trial (IALT), the National Cancer Institute of Canada Clinical Trials Group (NCIC CTG JBR.10) and the Adjuvant Navelbine International Trialist Association (ANITA), provide preliminary confirmation on the role of adjuvant chemotherapy, showing that platinum-based postoperative chemotherapy offers a significant 5-year survival benefit compared with surgery alone [3-5]. However, only 5%-15% of the individuals who received adjuvant chemotherapy achieved long-term survival improvement [6]. Therefore, it is very important to select subgroups of patients who are most likely to benefit from postoperative chemotherapy and who are potentially resistant to a given chemotherapy regimen. Recent studies have shown that expression of excision repair cross-complementation group 1 (ERCC1), ribonucleotide reductase subunit M1 (RRM1) and class III beta-tubulin (β-tubulinIII) could provide predictive value for patients treated with platinum agents, gemcitabine (GEMZ) and microtubule-interacting agents (including taxanes and vinorelbine (NVB)), respectively [7].

Topoisomerase II alpha (TopIIα) is a nuclear enzyme that catalyzes the conversion between DNA topological isomers and can be detected in cells with high proliferative activity. Many anticancer agents (i.e., anthracyclines) exert anticancer effects by stabilizing DNA cleavage and inhibiting DNA replication via binding and blocking the activity of TopIIα [8]. TopII-inhibiting chemotherapeutic agents, including irinotecan, etoposide and topotecan, are commonly used for the treatment of small cell lung cancer (SCLC), but they are seldom used for the treatment of NSCLC. However, it has been reported that one type of multidrug resistance (MDR) in lung cancer, atypical MDR, is mediated through an altered expression of TopII. This type of resistance was found to be associated either with decreased TopIIα expression or with a mutation that altered the interaction of the enzyme with the drug or DNA [9]. High TopIIα expression has been observed in many kinds of cancers, including breast cancer and NSCLC [10-13]. Although TopIIα expression and its implication for TopII inhibitors have been extensively investigated in several cancers, especially in breast cancer, few reports have described TopIIα expression and its cross resistance to other cytotoxic drugs in NSCLC [10,11,14-16]. In addition, some studies have reported that low levels of TopIIα gene or protein expression might be related with resistance not only to TopII inhibitors, such as etoposide and anthracycline, but also to other cytotoxic drugs, such as cisplatin, microtubule-interacting agents and gemcitabine, in some cancer cell lines or cancers [17-19]. However, little is known about the correlation between TopIIα expression, which can mediate cross resistance, and the effect of common chemotherapy drugs, including cisplatin, microtubule-interacting agents and GEMZ, in postoperative NSCLC.

To assess whether TopIIα might be a valuable biomarker in postoperative NSCLC patients who received chemotherapy, TopIIα expression levels in tumor samples were evaluated, and the prognostic and therapeutic predictive roles of TopIIα were analyzed. Ki67, another molecular marker that is related to proliferation, was evaluated as a control factor.

## Methods

### Patient data

Patients who underwent curative surgery for NSCLC between January 2004 and December 2007 received at least two cycles of adjuvant chemotherapy within two months after surgery in the General Hospital of Chinese People's Liberation Army (CPLA), and for whom a formalin-fixed, paraffin-embedded sample adequate for analysis was available, were included in the study. Patients who received neoadjuvant chemotherapy or neoadjuvant radiotherapy were excluded.

Data concerning clinicopathological features, including age, sex, smoking history, histological subtypes, pathological TNM stage, surgical methods, chemotherapy regimens and cycles, other treatments such as adjuvant radiotherapy, date of recurrence or metastasis, and survival information, were obtained by medical records, outpatient and telephone follow-ups. Outcome data including disease free survival (DFS) and overall survival (OS) were calculated. Clinical data were kept unavailable during immunohistochemistry (IHC). The study design and procedure involving the human tissue sample collection were reviewed and approved by the ethical board of the General Hospital of CPLA.

### Immunohistochemistry

Representative tumor paraffin-embedded blocks were selected and collected, after two pathologists reviewed, the tumor sample slices stained with hematoxylin and eosin (HE). Sections (4 μm thick) were cut from tumor specimens, placed onto 3-amino-propyltriethoxy-silane (APES) slides, and deparaffinized in xylene and gradient ethanol. Antigen retrieval was performed by placing slides in a high-pressure cooker in a 0.01 mM citrate buffer, pH 6.0, for 2.5 min at 100°C; they were then cooled for 20 min. Endogenous peroxidase activity was blocked by incubating the section in 3% H_2_O_2 _for 10 min, followed by rinsing in PBS solution three times. Immunohistochemical staining was performed with the two-step EnVision(tm)+ System Kit (Dako, Denmark). The sections were incubated with mouse anti-TopIIα monoclonal primary antibodies (clone 3F6, 1/75 dilution, Novocastra, UK) or mouse anti-Ki67 monoclonal primary antibodies (clone MIB1, 1/200 dilution, Dako) at 37°C for 60 min, followed by dextran polymer conjugated with horseradish peroxidase enzyme and secondary anti-mouse antibody (Dako). Slides were stained with 3,3'-diaminobenzidine tetrahydrochloride (DAB) chromogen and counter-stained with hematoxylin. Negative controls were conducted by adding PBS solution instead of the primary antibody, and positive controls were conducted by staining known-positive samples from our pathology specimen bank.

All the specimens were examined and scored by two independent pathologists without the knowledge of patient data, and their inter-observer concordance was over 90%. The number of positive cells in 1,000 tumor cells within 10 microscopic fields at ×200 magnification was counted and scored as follows: 0 (negative, <25%), 1 (focal, 25% to 49%), 2 (moderate, 50% to 74% ), 3 (diffuse, ≥75%). Samples with scores of 0 and 1 were considered to have low expression, whereas samples of 2 and 3 were considered to have high expression.

### Statistical analysis

Correlations between immunohistochemical expression and patient or tumor characteristics were performed by using the χ^2 ^test or Fisher's exact test, as appropriate. Survival curves were estimated by the Kaplan-Meier method, and differences in DFS among groups were compared by using the log-rank test. The COX proportional hazards model was used for multivariate analysis to assess the independent value of TopIIα expression. Two-sided P < 0.05 was considered statistically significant. All analyses were performed with the SPSS 16.0 software package.

## Results

### Patient characteristics

We enrolled 151 eligible patients (111 male and 40 female) in this study, who ranged in age from 31 to 81 years, with a median of 55 years, of whom 86 (60%) had a history of smoking. The main histological types included squamous cell carcinoma (41.0%), adenocarcinoma (39.1%), bronchioloalveolar carcinoma (10.0%), large cell carcinoma (6.6%) and adenosquamous (3.3%). Stage I, II and III were diagnosed in 35.8%, 31.1% and 33.1% patients, respectively. In addition, according to pathological T stage or N stage, T1, T2, T3 and T4 were diagnosed in 25.2%, 65.5% 3.3% and 6.0% patients, respectively, and N0, N1 and N2 were diagnosed in 41.1%, 33.1% and 25.8% patients, respectively. The majority had undergone lobectomy (81.5%). All patients received 2 to 8 cycles of adjuvant chemotherapy, of whom 114 (75.5%) finished at least 4 cycles. The main chemotherapy regimens included docetaxel (TXT, 26.5%), GEMZ (33.8%), NVB (26.5%) and paclitaxel (PTX, 13.2%) combined with or without cisplatin (DDP)/carboplatin (CBP). Of the 151 patients, 35 patients (23.2%), most of whom were diagnosed with stage III or N2, received adjuvant radiotherapy. Detailed characteristics are shown in Table 1.

**Table 1:** Clinical and pathological characteristics and univariate analysis for DFS

Factors		Number (%)	Median DFS (months)	P value
Sex	male	111 (73.5)	33.99	0.036*
	female	40 (26.5)	21.13	
Age	<55 years	74 (49.0)	21.45	0.330
	≥55 years	77(51.0)	31.97	
Smoking status	no	65 (43.0)	22.24	0.305
	yes	86 (57.0)	32.99	
Stage	I	54 (35.8)	NR^#^	0.001*
	II	47 (31.1)	33.64	
	III	50 (33.1)	15.18	
T stage	T1	38 (25.2)	NR^#^	0.03
	T2	99 (65.5)	18.76	
	T3-4	14 (9.3)	38.16	
N stage	N0	62 (41.1)	NR^#^	<0.001*
	N1	50 (33.1)	33.64	
	N2	39 (25.8)	13.37	
Histology	Adenocarcinoma	59 (39.1)	17.41	0.004*
	Non- adenocarcinoma	92 (60.9)	48.16	
Surgery method	Wedge resection	5 (3.3)	17.41	0.958
	Lobectomy	123 (81.5)	24.41	
	Pneumonectomy	23 (15.2)	31.15	
Chemotherapy regimens	Docetaxel-containing	40 (26.5)	18.60	0.523
	Vinorelbine- containing	40 (26.5)	48.16	
	Gemcitabine- containing	51 (33.8)	24.58	
	Paclitaxel- containing	20 (13.2)	24.41	
TopIIα	Low expression	117 (77.5)	19.38	0.012*
	High expression	34 (22.5)	NR	
Ki67	Low expression	96 (63.6)	22.24	0.517
	High expression	55 (36.4)	29.37	

* P < 0.05 was considered statistically significant; ^# ^NR = not reached

### TopIIα and Ki67 expression in NSCLC

**Figure 1:**
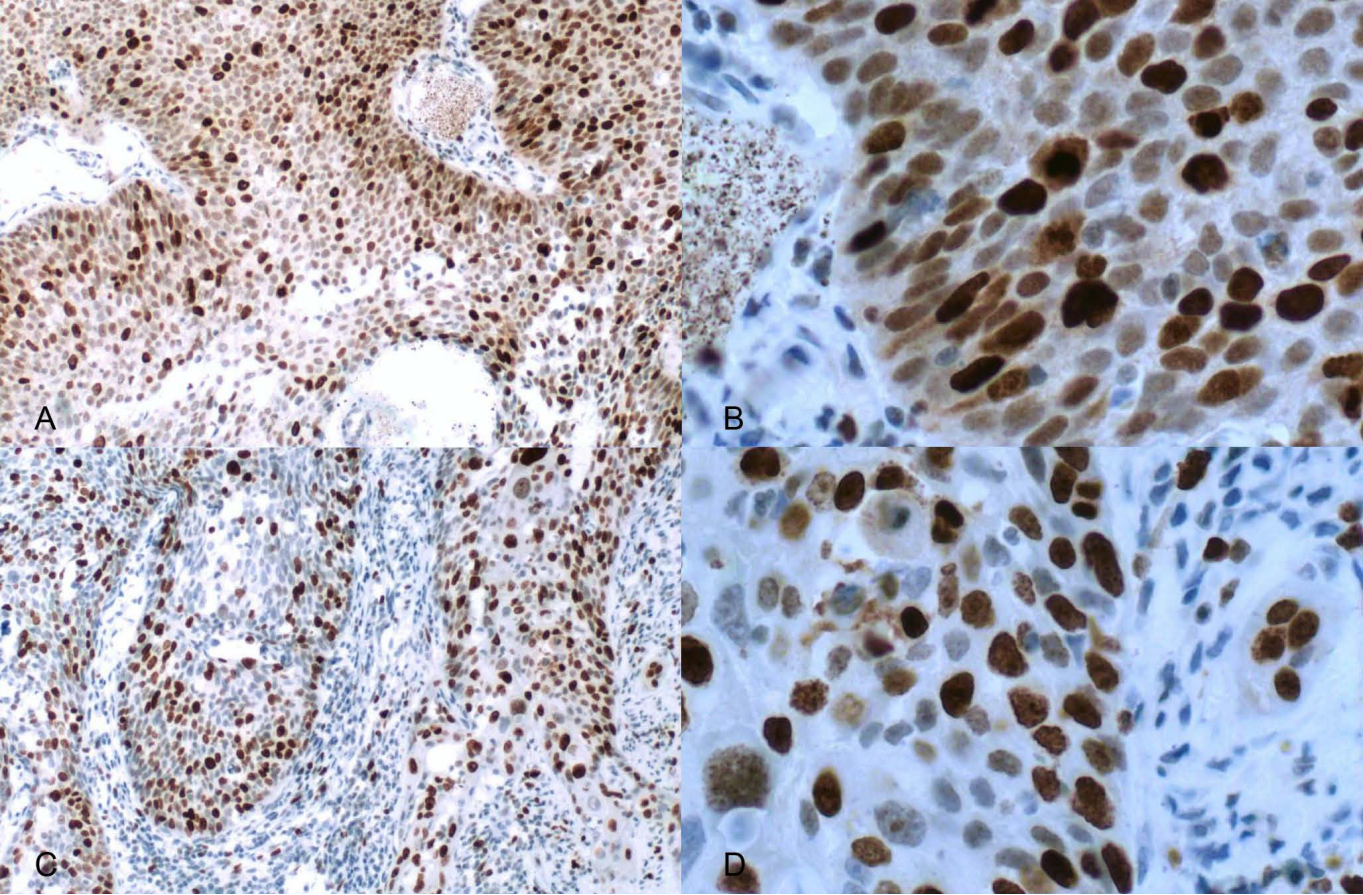
**Positive expression of TopIIα and Ki67 in non-small cell lung cancer by immunohistochemistry using the EnVision two-step method**. (A) and (B) correspond to ×200 and ×400 magnification of TopIIα respectively; (C) and (D) correspond to ×200 and ×400 magnification of Ki67 respectively

TopIIα (Figure 1A and 1B) and Ki67 (Figure 1C and 1D) were located in the nucleus. TopIIα and Ki67 were highly expressed in 22.5% and 36.4%, respectively, of all 151 patients. Additionally, the expression of TopIIα had a strongly positive correlation with that of Ki67 (r = 0.515, P < 0.001). Table 2 shows the correlation between TopIIα and Ki67 expression levels and clinical characteristics, including sex, smoking history, histological subtypes, pathological TNM stage, pathological T stage and pathological N stage.

**Table 2:** Association between TopIIα and Ki67 expression and clinical variables

Clinical variables	TopIIα expression (%)		P	Ki67 expression (%)		P
				
	High	Low		High	Low	
Sex						
Male	25 (22.5)	86 (77.5)	0.998	39 (35.1)	72 (64.9)	0.584
Female	9 (22.5)	31 (77.5)		16 (40.0)	24 (60.0)	
Smoking status						
No	16 (24.6)	49 (75.4)	0.591	25 (38.5)	40 (61.5)	0.651
Yes	18 (20.9)	68 (79.1)		30 (34.9)	56 (65.1)	
Histology						
Adenocarcinoma	14 (23.7)	45 (76.3)	0.775	22 (37.3)	37 (62.7)	0.860
Non- adenocarcinoma	20 (21.7)	72 (78.3)		33 (35.9)	59 (64.1)	
Stage						
I	10 (18.5)	44 (81.5)	0.177	13 (24.1)	41 (75.9)	0.034*
II	15 (31.9)	32 (68.1)		23 (48.9)	24 (51.1)	
III	9 (18.0)	41 (82.0)		19 (38.0)	31 (62.0)	
T stage						
T1	13 (34.2)	25 (65.8)	0.148	18 (47.4)	20 (52.6)	0.168
T2	18 (18.2)	81 (81.8)		34 (34.3)	65 (65.7)	
T3-4	3 (21.4)	11 (78.6)		3 (21.4)	11 (78.6)	
N stage						
N0	12 (19.4)	50 (80.6)	0.137	16 (25.8)	46 (74.2)	0.065
N1	16 (32.0)	34 (68.0)		23 (46.0)	27 (54.0)	
N2	6 (15.4)	33 (84.6)		16 (41.0)	23 (59.0)	

* P < 0.05 was considered statistically significant

### Patient outcomes and clinical predictors

After a median follow-up of 35.8 months (ranging from 16.4 - 63.7 months), 80 (53%) patients had metastatic or recurrent tumors and 40 (26.5%) patients had died. Causes of death were NSCLC (37 patients), radiation pneumonitis (1 patient), appearance of a secondary tumor (1 patient) and severe pulmonary infection (1 patient). The median DFS of all 151 patients was 24.3 months and the median OS was not reached. The primary purpose of the study was to assess the associations between clinical characteristics, TopIIα protein expression and efficacy of adjuvant chemotherapy. DFS was more representative than OS as an indirect measure of efficacy, because OS was often affected by treatments other than adjuvant chemotherapy after recurrence or metastasis. DFS was therefore used as the main outcome measure in this study.

**Figure 2:**
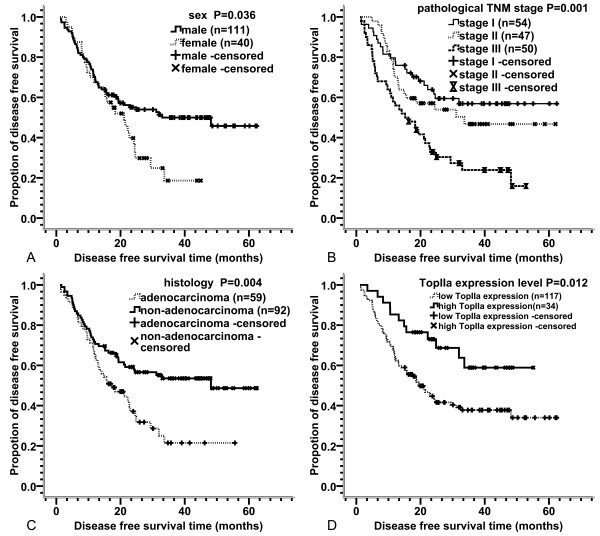
**DFS curves according to different clinical and pathological characteristics**. DFS curves according to: (A) sex; (B) pathological TNM stage; (C) histological subtype; (D) TopIIα expression level

Univariate survival analysis showed that sex, pathological TNM stage, pathological N stage, histological subtype (adenocarcinoma or not) and TopIIα expression level were prognostic factors related to DFS, while age, smoking history, surgical methods, pathological T stage, chemotherapy regimens and expression level of Ki67 were of no prognostic significance. Log-rank test indicated that male sex (P = 0.036, Figure 2A), earlier pathological TNM stage (P = 0.001, Figure 2B) and pathological N stage (P < 0.001), non-adenocarcinoma (P = 0.004, Figure 2C) and high TopIIα expression (P = 0.012, Figure 2D) predicted better DFS in postoperative NSCLC patients who received adjuvant chemotherapy. Although the P value for comparison of the survival curves in different T stages was less than 0.05 (P = 0.03), there was no significant difference between T1 stage and T3-4 stage (P = 0.677) or between T2 stage and T3-4 stage (P = 0.245) when pairwise comparisons of the three curves were performed. Only the difference between T1 stage and T2 stage was significant (p = 0.011). Detailed characteristics are also shown in Table 1.

### Correlations between TopIIα expression and the value of adjuvant chemotherapy

**Figure 3:**
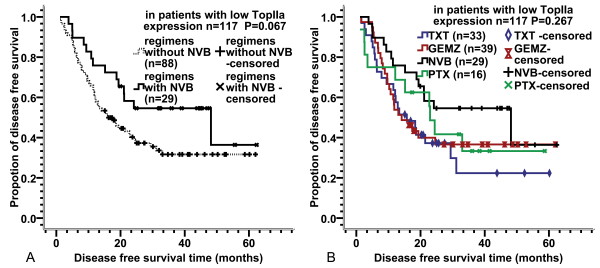
**Comparison of DFS rates among different chemotherapy subgroups in patients with low TopIIα expression**. (A) depicts comparison of DFS curves between the NVB regimen group and the group of other regimens without NVB; (B) depicts comparison of DFS curves among four chemotherapy subgroups

It was found that high expression of TopIIα was significantly correlated with better DFS in the patients studied, suggesting that patients with high TopIIα expression benefited more from the adjuvant chemotherapy than those with low TopIIα expression, and that the latter group of patients may be a potential chemotherapy-resistant population. Further stratified analysis of the correlation between the value of adjuvant chemotherapy regimens and TopIIα expression showed that when TopIIα was highly expressed, there were no significant differences in DFS between patients who received chemotherapy with or without TXT (P = 0.712), with or without NVB (P = 0.170), with or without GEMZ (P = 0.415), and with or without PTX (P = 0.763). In addition, when TopIIα expression was low, there were no significant differences in DFS between patients who received chemotherapy with or without TXT, with or without GEMZ, and with or without PTX (Table 3). However, in patients with low TopIIα expression, the median DFS was 48.16 months for those who received NVB-containing chemotherapy regimens and 16.23 months for those who received regimens without NVB (P = 0.065, Figure 3A). Although this difference was marginally insignificant, it is possible that NVB-containing adjuvant chemotherapy is better than others in patients with low TopIIα expression. To ascertain the predictive value of this observation with better certainty, the pooled comparisons as well as pairwise comparisons of DFS among four chemotherapy subgroups were investigated in patients with low and high TopIIα expression. In patients with high TopIIα expression, no significant differences in DFS were observed among the four chemotherapy subgroups regardless of pooled or pairwise comparisons (Table 4). In patients with low TopIIα expression, the P value was greater than 0.05 (P = 0.267, Figure 3B) for the pooled comparison of DFS among the four chemotherapy subgroups. Pairwise comparisons of the four survival curves showed no significant differences in P values between any two subgroups (Table 4 and Figure 3B), except the P value between the NVB and TXT subgroups. Pairwise comparison for low TopIIα expression indicated that the NVB-containing regimen was associated with better DFS than the TXT-containing regimen (P = 0.047).

**Table 3:** Comparison of DFS among different chemotherapy groups in patients with low TopIIα expression

Chemotherapy regimens		Number	Median DFS (months)	P
Vinorelbine	Containing	29	48.16	0.065
	Not containing	88	16.23	
Gemcitabine	Containing	39	14.13	0.530
	Not containing	78	22.74	
Docetaxel	Containing	33	15.74	0.194
	Not containing	84	22.74	
Paclitaxel	Containing	16	22.97	0.990
	Not containing	101	18.76	

**Table 4:** Pooled and pairwise comparisons of DFS among four chemotherapy regimens in patients with different TopIIα expression levels

TopIIα expression	Chemotherapy regimens	Vinorelbine (P)	Gemcitabine (P)	Docetaxel (P)	Paclitaxel (P)	P for each stratum
Low expression stratum	Vinorelbine		0.128	0.047*	0.322	0.267
	Gemcitabine	0.128		0.676	0.786	
	Docetaxel	0.047*	0.676		0.429	
	Paclitaxel	0.322	0.786	0.429		
High expression stratum	Vinorelbine		0.209	0.410	0.437	0.594
	Gemcitabine	0.209		0.895	0.902	
	Docetaxel	0.410	0.895		0.987	
	Paclitaxel	0.437	0.902	0.987		

* P < 0.05 was considered statistically significant

### Multivariate analysis for DFS

Multivariate analysis was performed with the COX proportional hazards model to explore whether the prognostic value of TopIIα would disappear when other common prognostic factors were considered. Correlation analysis showed that pathological T stage and pathological TNM stage (P < 0.001), and pathological N stage and pathological TNM stage (P < 0.001) were mutually associated. As pathological TNM stage is more widely accepted as a DFS-related parameter than pathological T stage and N stage, subsequent multivariate analysis was performed to incorporate sex, histology, pathological TNM stage and TopIIα expression level as covariates based on the significance of these factors in univariate analysis. The results from the Cox proportional hazard model using the forward stepwise method suggested that the histology subtype (adenocarcinoma vs. non-adenocarcinoma), TNM stage and the TopIIα expression level were independent prognostic factors of DFS in NSCLC (Table 5). In postoperative patients who received adjuvant chemotherapy, high TopIIα expression was a prognostic marker of longer DFS (hazard ratio 0.442, 95% confidence interval 0.239-0.818, P = 0.009), whereas adenocarcinoma (hazard ratio 2.140, 95% confidence interval 1.373-3.337, P = 0.001) and later TNM stage (hazard ratio 1.688, 95% confidence interval 1.291-2.208, P < 0.001) were related to a shorter DFS as compared to non-adenocarcinoma and earlier TNM stage.

**Table 5:** Multivariate analysis for factors related to DFS using the COX proportional hazard model

Factors	Wald	Hazard ratio	95% CI	P
Sex	0.903	0.784	0.474 - 1.295	0.342
Histology(adenocarcinoma)	11.277	2.140	1.373 - 3.337	0.001*
TNM stage (later)	14.631	1.688	1.291 - 2.208	<0.001*
Topoisomerase II alpha expression (high expression)	6.773	0.442	0.239 - 0.818	0.009*

* P < 0.05 was considered statistically significant

## Discussion

The results of this study suggest that high TopIIα expression in postoperative NSCLC patients who received adjuvant chemotherapy was associated with better DFS. However, other studies have reported that high TopIIα expression was associated with a poorer survival rate in other types of tumors, including hypopharyngeal carcinoma, breast cancer and salivary gland carcinoma [20-23]. The reason for this difference is probably that all patients in this study received adjuvant chemotherapy while no patient received it in other studies. We therefore postulate that adjuvant chemotherapy might overcome the adverse biology of cancers that express higher TopIIα protein. Additionally, other recent studies also showed that high expression or amplification of TopIIα promised a good response to chemotherapy, particularly to topoisomerase inhibitors, which are partly consistent with our study [20,24]. The results of our study imply that patients with high TopIIα expression might be able to obtain more benefits from adjuvant chemotherapy than those with low TopIIα expression. Similar results were found in Sève et al.'s study of the prognostic role of β-tubulinIII in postoperative NSCLC [25]. Sève et al. discovered that high expression of β-tubulinIII was related to poor survival in the absence of adjuvant chemotherapy, though adjuvant chemotherapy prolonged the relapse free survival (RFS) and OS in patients with high β-tubulinIII expression. Therefore, β-tubulinIII acted as not only a prognostic biomarker of DFS but also a predictor of response to adjuvant chemotherapy. To identify predictive factors for survival benefit from treatment, an untreated control group should be included in the study, because a good survival identified in a single-arm trial might simply result from prognostic factors within the cohort, and might not be a result of treatment [26]. However, the major limitation of this study was that patients who did not receive adjuvant chemotherapy were not included. Therefore, we can merely draw the conclusion that TopIIα acted as a prognostic biomarker of DFS in the postoperative NSCLC patients who received adjuvant chemotherapy. A further study including patients with and without adjuvant chemotherapy is required to clarify the predictive role of TopIIα with respect to the benefit of adjuvant chemotherapy. Moreover, TopIIα exhibited an independent significantly prognostic role for DFS, when common prognostic factors, including pathological TNM stage and histological subtype, were considered in multivariate analysis. In addition, the positive correlation between the expression of TopIIα and that of Ki67, as observed in this study, is consistent with prior published reports in NSCLC and breast cancer [13,27,28]. It suggests that higher TopIIα expression is associated with higher cell proliferation in NSCLC, as is the case with Ki67, which is a known cell proliferation marker. Although expression of Ki67 had a strongly positive correlation with expression of TopIIα, Ki67 was of no prognostic significance to DFS.

This is the first study reporting the evaluation of different adjuvant chemotherapy regimens by TopIIα expression level in NSCLC. We found that NVB-containing regimens were associated with better DFS than those without NVB in patients with low TopIIα expression, although the P value was marginally insignificant (P = 0.065). These results are concordant with previous reports related to the mechanism of atypical MDR, which is mediated by TopIIα showing that atypical MDR exhibited cross resistance to many anticancer drugs, except vinca alkaloids [29,30]. However, the result of pooled and pairwise comparisons of DFS between the four chemotherapy subgroups for low or high TopIIα expression showed that only the difference between the NVB and TXT subgroups was significant in patients with low TopIIα expression (P = 0.047). Therefore, it can be deduced that patients with low TopIIα expression are a possible MDR population and that NVB-containing regimens may be better than TXT-containing regimens in these patients. Although there is no sufficient evidence that the efficacy of NVB is better than that of GEMZ and PTX in patients with low TopIIα expression, DFS of the NVB subgroup was no worse than that of the GEMZ and PTX subgroups (Figure 3B). In addition, in patients with high TopIIα expression, there were no significant differences in DFS between the different regimens no matter what grouping or comparison methods were performed. Several studies have indicated that high TopIIα expression was a positive predictive factor for response to anthracycline-based chemotherapy in patients with primary breast cancer [31,32]. It is well known that anthracycline in an anti-topoisomerase drug, and should exert strong anti-cancer activity in patients with high TopIIα expression. However, further study is needed to determine whether an anti-topoisomerase-based adjuvant chemotherapy such as etoposide can really bring more benefits to postoperative NSCLC patients with high TopIIα expression than other chemotherapy regimens.

Owing to the limitations of retrospective research, the conclusions drawn from this study need to be further identified and validated in prospective clinical trials. Another deficiency of this study is that the median OS was not reached due to limited follow-up time. Considering that the connection between TopIIα expression and adjuvant chemotherapy was the main purpose of this study, DFS was more representative than OS for evaluating efficacy. Moreover, OS could be influenced by many factors in this retrospective study, which may possibly lead to a negative result for long-term survival. Prospective randomized clinical trials with larger sampling capacities and longer follow-up periods are needed to shed more light on the real relationship between TopIIα expression and the benefit from the given chemotherapy.

This study demonstrated a significant association between TopIIα expression and DFS, in which high TopIIα expression was associated with better DFS than low TopIIα expression, and NVB-containing adjuvant regimens were probably better than TXT-containing regimens in the low TopIIα expressors. Thus, TopIIα may be a good biomarker to predict the value of adjuvant chemotherapy, although further validation is required.

## Conclusions

In conclusion, TopIIα expression is an independent prognostic factor of DFS in postoperative NSCLC patients who received adjuvant chemotherapy. Low expression of TopIIα is related to poor DFS. NVB-containing adjuvant regimens are probably better than TXT-containing regimens in patients with low TopIIα expression. However, further investigation is needed to confirm the significance of TopIIα as a prognostic or predictive biomarker before these conclusions can be translated into clinical practice.

## Competing interests

The authors declare that they have no competing interests.

## Authors' contributions

SY collected the clinical data, performed immunohistochemical staining and statistical analyses, and drafted the manuscript. CL and WLX participated in the coordination of the study. LJ and LYL performed histological evaluation of immunohistochemical staining. JSC contributed to the conception and design of the study, interpretation of the data and revision of the manuscript. All authors have read and approved the final manuscript.

## Pre-publication history

The pre-publication history for this paper can be accessed here:

http://www.biomedcentral.com/1471-2407/10/621/prepub
